# Evaluation of different aesthetic indices for planning orthodontic treatment

**DOI:** 10.6026/973206300191394

**Published:** 2023-12-31

**Authors:** Subhash Chandra, Vivek Agarwal, Vinit Kumar Singh, Alok Kumar Gupta, Sandeep Singh, Shreya Koleshwar, Ramanpal Singh Makkad

**Affiliations:** 1 Department of Orthodontics and Dentofacial Orthopedics, Dental College, RIMS, Ranchi, Jharkhand, India; 2 Department of Orthodontics and Dentofacial Orthopedics, Haldia Institute of Dental Sciences and Research, Haldia, West Bengal, India; 3 Department of Orthodontics and Dentofacial Orthopedics, Vananchal Dental College, Farathiya Garwha, Jharkhand, India; 4 Department of Orthodontics and Dentofacial Orthopedics Vananchal Dental College, Farathiya Garwha, Jharkhand, India; 5 Department of Orthodontics and Dentofacial Orthopedics, Terna Dental College and Hospital, Navi Mumbai, Maharashtra, India; 6 Department of Orthodontics and Dentofacial Orthopedics, Government Dental College and Hospital, Chh. Sambhajinagar, Maharashtra, India; 7 Department of Oral medicine and Radiology, New Horizon Dental College and Research, Institute, Sakri, Bilaspur, Chhattisgarh, India

**Keywords:** Aesthetic indices, orthodontic treatment, planing

## Abstract

Three aesthetic indices namely aesthetic component of index of orthodontic treatment needs (IOTN -AC), dental aesthetic index (DAI) and dental aesthetics screening index (DESI) were compared in orthodontic treatment. 242 participants (160 female and 82 male) who were interested in orthodontic treatment participated. The individuals' ages ranged from 16-25 years. Three aesthetic indices namely IOTN -AC, DAI and DESI were evaluated for each participant. The overall accuracy of DAI, AC-IOTN and DESI in assessment of dental aesthetics in orthodontic treatment was 62%, 68% and 64% respectively. The Negative predictive value (NPV) was higher than Positive predictive value (PPV) for all indices. The sensitivity was greater than specificity for all indices. It was observed that values of sensitivity, specificity, PPV and NPV were high for AC-IOTN. The findings were significant statistically (p<0.05).

## Background:

Over the last three decades, there has been a significant surge of requests for orthodontic therapy [1, 2]. The social science studies show that an unsatisfactory dental appearance can stigmatize, obstruct professional development and community acceptance, promote discrimination, and negatively impact self-concept provide the basis for recommendations for treatment based on aesthetic impairment [3,4]. Since orthodontic issues are typically not linked to severe morbidity or mortality, most medical practitioners tend to disregard them as less significant [5,6]. On the other hand, research indicates that malocclusions significantly damage the afflicted person's mental well-being [7, 8]. Investigators in epidemiological investigations of malocclusion disagreed much for a long time, particularly on the acceptable degree of variation from the ideal within the parameters of normalcy [9, 10]. The aesthetic component (AC) of the index of orthodontic treatment need (IOTN) was used to determine the study group's requirement for orthodontic therapy [11,12]. It is frequently utilised in dental epidemiological studies to estimate the probable socio-psychological impacts of each the individual's malocclusion and to prioritize orthodontic therapy [13,14]. Because malocclusion is so common and has such negative functional and psychological effects, it is often listed as the third most important oral health concern in the world [15,16]. A few effects of malocclusion include impaired dental appearance that affects social relationships, confidence, and psychological health, bite including phonetic issues, and temporo-mandibular instability [17,18]. The Dental Aesthetic Index (DAI) was introduced in 1986 and has been widely utilised in multiple epidemiological investigations for assessing the incidence of malocclusion associated orthodontic therapy requirements [1- 7]. While other indices can determine the severity of malocclusion to varying degrees, the DAI has been frequently employed. In fact, the World Health Organization (WHO) listed it as a suggested technique for evaluating malocclusion within a year after it was published [3-8]. The DAI relies on a mathematical formula that adds together the values of occlusal parameters related to malocclusion (missing teeth, over jet, irregularity, crowding, open-bite, molar class and spacing, in order to provide a score[1- 6]. The DAI offers a wealth of information about the kind and degree of malocclusion and is dependable, impartial, and simple to utilize [4-7]. Due to its diagnostic and educational applications, the DAI equation is convenient despite having significant drawbacks, including inadequate recognition of cross bite and deep bite malocclusions [14-18]. The DAI is useful for determining the most appropriate treatment in addition to gauging the extent of malocclusion and its effects on dental wellness in the community [19,20, 21,22,23]. Textbooks and expert articles are the main sources of widespread understanding on dental aesthetics, and these are widely accepted in Western countries Ideal dento-facial parameter measurements are given there, with the assumption that discrepancies could lead to a deficiency in aesthetic quality [11-14]. Nevertheless, not much information is presented regarding the tenets that underpin these professional judgements. Perceptions of aesthetics are likewise highly subjective [15-18]. Every dental professional has probably noticed that patients' and dentists' perceptions of dento-facial aesthetics can differ greatly. Dento-facial aesthetics measurement is a difficult undertaking that requires an extensive index that can achieve it [12-17]. Crucial aesthetic parameters are required for objective measurement [12,17, 18]. These parameters include the width-to-height proportions of the maxillary anterior teeth, incisor angulations, papilla height, root coverage, gingival contour, smile line, location of the dental and facial midline and lip line. They developed the "Dental Aesthetic Screening Index" (DESI), which consists of a total of 5 extraoral along with 7 intraoral items, based on these results [12]. The DESI classifies the aesthetic result using a cumulative score and a rating scale of five points for each item's quantitative evaluation. Excellent aesthetics are represented by a low sum rating, whereas bad aesthetics are represented by a high sum rating [12,15, 18,19]. Application of DESI in orthodontic treatment and its comparison with IOTN - AC and DAI is not available. Therefore, it is of interest to compare three aesthetic indices namely IOTN -AC, DAI and DESI in orthodontic treatment.

## Methods and Materials:

A cross-sectional analytical investigation was carried out from June 2023 to December 2023 in study participants seeking orthodontic treatment before orthodontic treatment began. There were 242 participants in the sample for the present investigation (160 female and 82 male). The individuals' ages ranged from sixteen to twenty-five. The overall sample size's mean age was 18.49 ± 2.04 years. The average age of the male and female respondents was 18.10 ± 2.08 and 18.64 ± 2.07 years, respectively. There was evaluation of each study participants using three aesthetic indices namely IOTN -AC, DAI and DESI.

## IOTN-AC:

Patients were asked to give a score for their condition using the IOTN-AC scale in order to gauge how they felt about themselves. In the present investigation, there were no interviews done to assess patient perspectives. Following the gathering of orthodontic documentation at the preceding appointment, participants were provided with their pretreatment monochrome intra-oral frontal images during their subsequent visit for banding and bonding. The lead investigator used Microsoft Office Picture Manager to edit the intra-oral frontal photographs, which had been captured by the orthodontic trainees at the dental clinic, to ensure consistency in magnification, color and size (converting color photos to monochrome). Printouts of the photos were presented to the patients at their chairs, and the scores were determined using the IOTN-AC standard. Concurrently with the patients at the chair side, the orthodontist also recorded the conditions. In order for the orthodontist and patient to record their scores on different data sheets at the same time, the patients were instructed to notify one another when they were prepared to grade their conditions. Utilising pretreatment research cast models, the orthodontist used the IOTN ruler to evaluate the IOTN-DHC and find the maximum number for the extent of malocclusion.

## DAI:

For the investigation, high-quality diagnostic replicas with permanent teeth were chosen. One examiner, who had received training from an experienced specialist, examined the models. The DAI score has been calculated by multiplying the total of 10 components by their respective weights and then adding a constant of 13. A periodontal probe with a millimeter scale was used to take the readings. On a previously created spreadsheet, the results were gathered. A programme that facilitates data organization on spreadsheets along with tabulated calculations computed the final result quickly. The models have been evaluated twice, separated by seven days, in order to remove bias.

## DESI:

It has seven intraoral and five extra-oral items, along with grading scales for recording the cumulative results for the overall evaluation (12-60 points), intraoral (7-35 points), and extra-oral (5-20 points). Excellent aesthetics are represented by a low sum score, whereas bad aesthetics are represented by a high sum score. Five-point rating systems are used for quantification [12]. Three professors with at least ten years of clinical and professional experience and expertise in the field of orthodontics established the gold standard for the necessity of orthodontic treatment [17]. They looked at each of the 242 study participants' photos and study models independently. Based on the results of a clinical assessment, each model and set of images was coded as "no requirement for orthodontic therapy," "optional orthodontic therapy," or "orthodontic therapy required." The researchers discussed the differences in their assessments of the models in order to get to a consensus.

## Statistical analysis:

The features of the sample were described using the frequency distribution. Pearson's Chi-square test was used to assess differences across age groups. For each age group, the mean DAI scores and the frequency of malocclusion as a function of DAI score were determined. The Student's t-test was utilised to compare the means of the continuous variables. Using the Kolmogorov-Smirnov test, the normality of the variable "time" was assessed. The Wilcoxon test was used to compare the amount of time required to analyse the indices. For every analysis, a significance level of 5% was chosen. Sensitivity, specificity, accuracy, negative predictive value (NPV), and positive predictive value (PPV) were all calculated as part of the indices validation process.

## Results:

The time taken for carrying out assessment was 119.0 ±35.2 seconds, 61.5 ±24.8 seconds and 68.6 ±30.9 seconds in DAI, AC-IOTN and DESI respectively. The duration was low in making assessment using AC-IOTN and maximum in DAI. The time duration in DESI was lesser than DAI but greater than AC-IOTN. The findings were significant statistically (p=0.021) (Table 1). According to data obtained, 88% study participants according to DAI needed orthodontic treatment, while 12% didn't required such treatment. On the other hand, 91% study participants according to AC-IOTN needed orthodontic treatment, while 9% didn't required such treatment. It was found that 89 % study participants according to DESI needed orthodontic treatment, while 11 % didn't required such treatment. The percentage of study participants needing orthodontic treatment was high according to AC-IOTN while it was low according to DAI. The values of DESI is less than AC-IOTN but greater than DESI. The findings were significant statistically (p=0.003) (Table 2). The overall accuracy of DAI, AC-IOTN and DESI in assessment of dental aesthetics in orthodontic treatment was 62%, 68% and 64% respectively. The NPV was higher than PPV for all indices. The sensitivity was greater than specificity for all indices. It was observed that the values of sensitivity, specificity, PPV and NPV were high for AC-IOTN. The findings were significant statistically (Table 3).

## Discussion:

The findings were similar to some previous studies which showed better accuracy, sensitivity, specificity for IOTN-AC in assessment for need for orthodontic treatment based on dental aesthetic assessments [16-19]. However, there were some studies which have findings not similar to our study. These studies showed that DAI is more accurate and have high sensitivity and specificity in assessment of need for orthodontic treatment by assessment of dental aesthetics [1- 6]. In Western countries, textbooks and scholarly publications are the primary sources of commonly accepted knowledge on dental aesthetics [23,24]. There are ideal measurements for dento-facial parameters, based on the theory that differences may result in a lack of aesthetic quality. However, little information about the principles guiding these expert opinions is provided. Aesthetic perceptions are also extremely personal. Any dental practitioner worth their salt has undoubtedly observed that opinions on dento-facial aesthetics can vary widely across dentists and patients [25,26]. Dento-facial aesthetics measurement is a challenging task that needs a comprehensive index to be accomplished [12, 13]. A group of scientists identified which essential aesthetic factors are necessary for objective measurement after reviewing the literature [9-12]. These characteristics include the maxillary front teeth's width-to-height ratios, incisor angulations, papilla height, root coverage, gingival contour, smile line, and locations of the lip line and dental and facial midlines [13- 16]. Based on these findings, they created the "Dental Aesthetic Screening Index" (DESI), which consists of a total of 5 extraoral and 7 intraoral items[10-12].T he DESI uses a five-point scoring system for each item's quantitative evaluation and a cumulative score to classify the aesthetic outcome. A low sum rating denotes excellent aesthetics, while a high sum rating denotes poor aesthetics [11- 14]. The findings were similar to some previous studies which showed lesser time taken for IOTN-AC in assessment for need for orthodontic treatment based on dental aesthetic assessments [17, 21]. However, there were some studies which have findings not similar to our study. These studies showed that DAI require less time in assessment of need for orthodontic treatment by assessment of dental aesthetics [2- 6]. Since its introduction in 1986, the Dental Aesthetic Index (DAI) has been extensively used in numerous epidemiological studies to evaluate the prevalence of malocclusion and the need for orthodontic care [13- 16]. Although there are several indices that can assess malocclusion severity to a different extent, the DAI has been widely used. In fact, a year after its publication, the World Health Organization (WHO) included it on their list of recommended methods for assessing malocclusion [1-4]. The DAI uses a mathematical formula to provide a score by adding the values of occlusal factors (missing teeth, overjet, irregularity, crowding, open-bite, molar class, and spacing) associated to malocclusion. The DAI is a trustworthy, unbiased, and user-friendly resource that provides a plethora of information regarding the kind and severity of malocclusion [15- 18]. The DAI equation has substantial limitations, such as poor identification of cross bite and deep bite malocclusions, but it is nevertheless practical because of its diagnostic and instructional uses. The DAI is helpful in assessing the degree of malocclusion and its impact on community dental wellness, as well as in deciding on the best course of action [19-22]. The findings were similar to some previous studies which found greater percentage of participants needing orthodontic treatment while using IOTN-AC in assessment for need for orthodontic treatment based on dental aesthetic assessments [18-24]. However, there were some studies which have findings not similar to our study. These studies found greater percentage of participants needing orthodontic treatment while using DAI in assessment of dental aesthetics [1-7]. For a very long time, researchers in epidemiological studies of malocclusion couldn't agree much, especially on how much deviation from the ideal was acceptable within the bounds of normalcy [13-14]. The study group's need for orthodontic therapy was assessed using the aesthetic component (AC) of the indicator of orthodontic treatment need (IOTN). It is often used in dental epidemiological studies to prioritize orthodontic therapy and determine the likely socio-psychological effects of each patient's malocclusion [15-19]. Malocclusion is frequently ranked as the third most significant oral health issue in the globe due to its widespread occurrence and detrimental functional and psychological implications. A few consequences of malocclusion include bite problems, including phonetic difficulties, temporo-mandibular instability, and poor dental appearance that impacts social interactions, confidence, and psychological health [18-21]. Treatment suggestions based on aesthetic impairment are based on social science research demonstrating the stigmatizing effects of an unacceptable dental look, as well as how it can hinder professional development and community acceptability, encourage discrimination, and negatively impact self-concept [22,23]. Because orthodontic problems are usually not associated with major morbidity or mortality, most physicians consider them to be less important. However, studies show that malocclusions seriously impair the mental health of the affected individual [24,25]. For a very long time, researchers in epidemiological studies of malocclusion couldn't agree much, especially on how much deviation from the ideal was acceptable within the bounds of normalcy [23-26].

## Conclusion:

AC-IOTN is useful for analyzing dental aesthetics in patients seeking orthodontic treatment and it is a potent instrument for patient counselling and scheduling.

## Figures and Tables

**Table 1:** Data showing time taken in assessment using DAI, AC-IOTN and DESI

	**DAI**	**AC-IOTN**	**DESI**
Average time (SD)	119.0 (±35.2)	61.5 (±24.8)	68.6 (±30.9)
Median time *	117	48	49
Range (Minimum-Maximum)	47.0-216.0	4.0-201.1	5.0-207.0
P value	0.021*		

**Table 2:** Comparison of need of orthodontic treatment according to DAI, AC-IOTN and DESI

	**Need of treatment**	**No Need of treatment**
DAI	88%	12%
AC-IOTN	91%	9%
DESI	89%	11%
P value	0.003*	

**Table 3:** Properties of three esthetic indices according to golden standards

	**Sensitivity (%)**	**Specificity (%)**	**PPV (%)**	**NPV (%)**	**Accuracy (%)**
DAI (CI95%)	92 (82-97)	15 (9-27)	29 (25-33)	83 (52-96)	62 (52-72)
AC-IOTN (CI95%)	96 (91-98)	20 (11-32)	32 (28-35)	97 (88-99)	68 (58-78)
DESI (CI95%)	94 (89-96)	18 (9-28)	30 (25-33)	95 (86-97)	64 (56-72)
P value	0.0021*	0.0034*	0.0012*	0.0044*	0.001*

## References

[1] Balachandran P (2021). J Oral BiolCraniofac Res..

[2] Fernández-Riveiro P (2021). Int J Environ Res Public Health..

[3] Boronat-Catalá M (2016). J Orthod..

[4] Cardoso CF (2011). Int J Environ Res Public Health..

[5] Evans R (1987). Eur J Orthod..

[6] Nagalakshmi S (2017). J Indian Soc Pedod Prev Dent..

[7] Jenny J, Cons NC (1996). Am J OrthodDentofacial Orthop..

[8] Grzywacz I (2003). Eur J Orthod..

[9] Hunt O (2002). Eur J Orthod..

[10] Birkeland K (1996). Am J Orthod Dentofacial Orthop..

[11] Kok YV (2004). J Orthod..

[12] Frese C (2019). J Esthet Restor Dent..

[13] Danaei SM (2007). Rev Santé Méditerranée Orient..

[14] Abdullah MS (2001). J Community Dent Health..

[15] Dawjee SM (2002). J S Afr Dent Assoc..

[16] De Oliveira CM (2003). Brit Dent J..

[17] Freer E, Freer TF (1999). Aust. Orthod. J..

[18] Batarrita JA (2005). Gac. Sanit..

[19] Ovsenik M (2007). Am. J. Orthod. Dentofac. Orthop..

[20] Sim J (2005). Phys. Ther..

[21] Beglin FM (2001). Am. J. Orthod. Dentofac. Orthop..

[22] Lima RB (2010). Cien. Saude Colet..

[23] Arruda Am (2008). J. Orthod. Dentofac. Orthop..

[24] Firestone AR (2002). Am. J. Orthod. Dentofac. Orthop..

[25] Younis JW (1997). Community Dent. Oral. Epidemiol..

[26] Soumes M (2006). Eur. J. Orthod..

